# The emerging role of nutritional support in the supportive care of pediatric patients undergoing hematopoietic stem cell transplantation

**DOI:** 10.3389/fnut.2023.1075778

**Published:** 2023-02-15

**Authors:** Edoardo Muratore, Davide Leardini, Francesco Baccelli, Francesco Venturelli, Sara Cerasi, Andrea Zanaroli, Marcello Lanari, Arcangelo Prete, Riccardo Masetti, Daniele Zama

**Affiliations:** ^1^Pediatric Oncology and Hematology “Lalla Seràgnoli”, Istituto di Ricovero e Cura a Carattere Scientifico, Azienda Ospedaliero-Universitaria di Bologna, Bologna, Italy; ^2^Department of Experimental, Diagnostic and Specialty Medicine (DIMES), University of Bologna, Bologna, Italy; ^3^Pediatric Emergency Unit, Istituto di Ricovero e Cura a Carattere Scientifico, Azienda Ospedaliero-Universitaria di Bologna, Bologna, Italy; ^4^Department of Medical and Surgical Sciences (DIMEC), University of Bologna, Bologna, Italy

**Keywords:** HSCT, nutritional support, pediatric oncology, gut microbiome, supportive care

## Abstract

Allogeneic Hematopoietic Stem Cell Transplantation (allo-HSCT) represents a potentially curative strategy for many oncological, hematological, metabolic, and immunological diseases in children. The continuous effort in ameliorating supportive care represents one of the cornerstones in the improvement of outcome in these patients. Nowadays, more than ever nutritional support can be considered a key feature. Oral feeding in the early post-transplant period is severely impaired because of mucositis due to conditioning regimen, characterized by, mainly by vomiting, anorexia, and diarrhea. Gastrointestinal acute graft-versus-host-disease (GvHD), infections and associated treatments, and other medications, such as opioids and calcineurin inhibitors, have also been correlated with decreased oral intake. The consequent reduction in caloric intake combined with the catabolic effect of therapies and transplantation-related complications with consequent extended immobilization, results in a rapid deterioration of nutritional status, which is associated with decreased overall survival and higher complication rates during treatment. Thus, nutritional support during the early post-transplantation period becomes an essential and challenging issue for allo-HSCT recipients. In this context, the role of nutrition in the modulation of the intestinal flora is also emerging as a key player in the pathophysiology of the main complications of HSCT. The pediatric setting is characterized by less evidence, considering the challenge of addressing nutritional needs in this specific population, and many questions are still unanswered. Thus, we perform a narrative review regarding all aspects of nutritional support in pediatric allo-HSCT recipients, addressing the assessment of nutritional status, the relationship between nutritional status and clinical outcomes and the evaluation of the nutritional support, ranging from specific diets to artificial feeding.

## Introduction

Hematopoietic stem cell transplantation (HSCT) is a mainstay in the treatment of a variety of hematological, oncological, and immunological diseases of childhood (1). Despite often representing the only curative treatment, it is hampered by high mortality and morbidity rate due to infective and immune-mediated complications (2, 3). Intensive supportive care is provided to patients undergoing HSCT to guarantee broader applicability of the procedure. In recent years, nutritional support has been a subject of growing interest within this field.

Indeed, pediatric patients undergoing HSCT receive a conditioning regimen including high doses of chemotherapy and/or total body radiation in a short time frame, which produce detrimental effects on the gastrointestinal (GI) system (4). Moreover, graft-versus-host disease (GvHD), an immune-mediated complication caused by the activation of donor T lymphocytes, can directly involve the GI system resulting in profuse diarrhea and malabsorption (2). These complications impair patients’ nutritional status, characterized by severe weight loss and malnutrition (5).

The importance of nutritional status in pediatric patients undergoing HSCT has been well established, both in terms of HSCT-related outcomes and for the long-term consequences on development (6). Moreover, nutrition has been shown to be a strong modulator of the gut microbiota (GM), the ecosystem composed of bacteria, viruses, and fungi that primarily live in our GI system. Notably, recent evidences have associated GM diversity and composition with HSCT clinical outcomes, suggesting the possibility to use diet as a GM modulator (7, 8). Nutritional support during HSCT includes the type of diet administered during the conditioning chemotherapy, the nutritional support during the neutropenic phase, and the diet after the re-alimentation and after the discharge. Each period is characterized by different nutritional needs and challenges to face. Although nutritional support is being increasingly considered a non-secondary element in supportive therapy, only a little evidence is available, particularly for pediatric patients. Recommendations for the management of nutritional needs in pediatric cancer patients have recently been published (9); however, recommendations with a specific focus on pediatric HSCT recipients are lacking.

In this article, we aim to comprehensively review the available evidence on nutritional support for pediatric patients undergoing HSCT. We will discuss the two-sided relationship between HSCT and nutritional status and the evidence available on the type of nutrition in the different phases of transplant. In particular, we will address the assessment of nutritional status, the relationship between nutritional status and clinical outcomes and the evaluation of nutritional support, ranging from specific diets to artificial feeding.

## Impact of HSCT on nutrition and nutritional status

Malnutrition affects 10–50% of children undergoing HSCT and it has a complex and multifactorial nature (10). As briefly mentioned, pediatric allo-HSCT recipients receive a conditioning regimen, which includes high-dose chemotherapy and/or total body irradiation. Common side effects of this treatment include oral and/or enteral mucositis and other gastrointestinal *sequelae*, such as vomiting, anorexia, and diarrhea (11, 12). As a result, oral intake is significantly impaired and it declines rapidly in the first few days after treatment: Data shows that during the first eight days after treatment most patients introduce less than 60% of their estimated energy requirements (12, 13).

On top of these common conditioning regimen side effects, some complications of allo-HSCT, such as gut aGvHD and infections, can contribute to furtherly reduce oral intake.

Moreover, certain medications that may be administered to these patients as supportive treatments, such as opioids, or immunosuppressive treatments, such as calcineurin inhibitors, can cause anorexia, nausea, vomiting, decelerated bowel movements and dysgeusia, which is particularly associated to cyclosporin. All these side effects have a negative impact on these children’s nutritional status and contribute to reduce their tolerance to oral feeding (14).

At the same time, due to their therapy regimen, allo-HSCT patients experience relevant metabolic changes: most of the data claims that their basal metabolic rate is increased by an estimated 30–50% (15) and a systemic inflammatory syndrome is frequently activated. This inflammation has variable intensity, but it has impacts on several metabolic pathways: Protein metabolism, with altered protein turnover, loss of muscle mass and increased production of acute phase proteins; carbohydrate metabolism, with insulin resistance and impaired glucose tolerance; and lipid metabolism, with loss of fat mass (11). In addition, long cycles of therapies imply prolonged bed-confinement times, which contributes to furtherly shrink muscle mass. Furthermore, it must be considered that by the time that pediatric oncological patients begin HSCT treatment protocols, they most likely have already undergone multiple cycles of chemotherapy and thus already have a compromised nutritional status.

The global reduction in caloric intake combined with the metabolism-accelerating (or catabolic) effect of chemotherapies and with the transplantation-related complications may result in a severe deterioration of nutritional status. Moreover, electrolyte disturbances often happen due to conditioning regimen, antimicrobial drugs, GvHD prophylaxis, impaired renal function and altered nutrition, and need frequent observation and corrections by nutritional support (16).

Over the past years, reduced intensity and non-myeloablative conditioning regimens have been developed in order to reduce their toxicity on allo-HSCT recipients. Nonetheless, considering the complex and multifactorial nature of malnutrition and the on-going controversy over the methods of nutritional interventions, many patients continue to experience malnutrition, hence an improvement in supportive care modalities is becoming essential (17).

## Assessment of nutritional status in pediatric transplanted patients

Even if malnutrition in children with cancer can significantly affect outcomes, it continues to be largely unrecognized and unmonitored, and very few studies have examined nutritional assessment in children undergoing HSCT (18, 19). As the ESPEN guidelines underline, it is important to screen patients evaluating nutritional intake, weight change and body mass index (BMI) at diagnosis and repeated depending on the stability of the clinical situation, and then, in patients with abnormal screening, perform an objective and quantitative assessment of nutritional intake, nutrition impact symptoms, muscle mass, physical performance and the degree of systemic inflammation (11). In adults, the gold standard for the evaluation of the nutritional status in oncology is the PS-SGA (Patient-Oriented Subjective Global Assessment) score (20), which is divided into two parts: One filled in by the patient about subjective sensations on food intake, weight loss perception, nausea, vomiting, dysgeusia, performance status, and one filled in by the dietitian with anthropometric and clinical data. Lacking validated instruments for nutritional assessment in patients undergoing HSCT, this tool could reasonably be applied to transplanted patients too, and possibly validate by a specific research (20). In children screening tools such as SCAN (nutrition screening tool for childhood cancer), which considers information like the type of cancer, the intensity of treatment, the presence of GI symptoms, the food intake over the past week, the weight loss over the past month, and the presence of signs of undernutrition, have been developed over time (18, 19). These screening tools use different anthropometric parameters. Indeed, BMI has conventionally been used to determine body habitus, but it doesn’t discriminate between adipose tissue and muscle, and can be influenced by the hydration status, thus other anthropometric measures can more accurately determine nutritional status (19, 21). Mid-upper arm circumference (MUAC) is a sensitive parameter to detect the risk of malnutrition in children undergoing HSCT, as it provides a good projection of whole-body muscle and fat mass (19). It is important to plot both these parameters on growth charts according to age and gender to determine the Z-score (22). DEXA (Dual Energy X-ray Absorptiometry), based on the different absorption of two peaks of x-rays by the soft tissue and the bone, represents the current clinical gold standard for bone and body composition, as it gives accurate measures of whole-body fat mass, lean body mass and bone mineral content, even if it does not discern visceral from subcutaneous fat (20). BIA (Bioelectrical Impedance Analysis), which evaluates cellular electrical properties, being based on the principle that all membrane electrical properties are influenced by changes in cell mass which is in turn dependent on metabolic rates and diet, measures total body water, fat mass and fat-free mass and has demonstrated to be useful in children undergoing HSCT as well (23).

**FIGURE 1:**
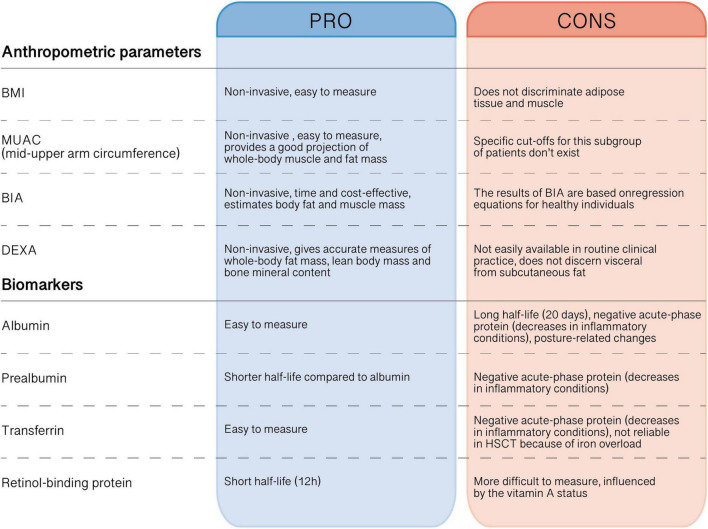
Advantages and disadvantages of anthropometric and biochemical parameter used to assess the nutritional status in pediatric HSCT recipients.

To better measure malnutrition, several biomarkers have been investigated, which should be used in conjunction with these tools. The best indices have to be cheap, easy to evaluate and independent from parameters of inflammation like acute phase proteins (24, 25). In a recent review conducted on studies on adults, no reliable biomarker has been identified as the gold standard for the assessment of nutritional status in patients undergoing HSCT, even if these biomarkers, together with other exams such as glycemia, electrolytes, lipid profile, vitamins and trace elements, are normally used in the clinical practice to monitor nutritional therapy and correct any nutritional deficiency (20). The biomarkers that have been considered include anabolic proteins such as albumin and prealbumin, retinol-binding protein (RBP), and transferrin (TRF). Albumin and prealbumin, also named transthyretin, have traditionally been used as markers of the nutritional status of patients. Prealbumin is preferred because of its shorter half-life (2–3 days) and thus can be applied to assess the short-time effectiveness of nutritional support (26). Indeed, albumin has a half-life of 20 days so a decrease in its concentration is related to long periods of nutritional deficit, while prealbumin reflects more acute changes of the nutritional state (24). However, they both are negative acute-phase proteins, thus they decrease in inflammatory conditions like cancer. Transferrin seems to be a useful malnutrition biomarker too, with a half-life of approximately 10 days, but it also is a negative acute-phase protein and, in addition, it is not reliable in HSCT, because patients often receiving several blood transfusions and present an iron overload (20, 26). Retinol-binding-protein represents the anabolic protein with the shortest half-life (12 h), but it is more difficult to measure and it is influenced by the vitamin A status (26). Even if some studies suggest that these biochemical indices are not sufficiently reliable because inflammation leads to depression of all protein synthesis, thus when acute-phase protein levels are high these proteins lose their function as parameters detecting malnutrition, Rzepecki et al. showed that these biomarkers could be helpful in adults in specific patients treated with HSCT for nutritional assessment (25). Other parameters proposed as biomarkers in transplanted patients include total urinary nitrogen, total plasma proteins, citrulline and IGF-1, but more studies are needed in order to validate them (20). In Figure 1, we summarized the different methods of nutritional assessment, highlighting the main advantages and disadvantages of each method.

## The relationship between nutritional status and clinical outcomes

Several factors are known to influence HSCT recipients’ outcome. Firstly, the type of the underlying disease and its stage, the presence of other comorbidities, the type of stem cell transplantation and HLA mismatch, the stem cell source, and the patient’s age. In contrast to these factors, that are disease-specific and non-modifiable, nutritional status can potentially be improved through adequate nutritional support (14). In adult transplant recipients, several studies highlighted how nutritional status can influence allo-HSCT outcome. Serum albumin deficiency prior to transplant is associated with increased non-relapse mortality (15), whereas malnutrition, defined by both low BMI and weight loss before and during transplantation, is considered a risk factor for severe aGvHD (27) and overall mortality (28). On the other side, conflicting results are reported regarding the relationship between obesity and clinical outcome, but the majority of studies point toward a net negative effect (29). In pediatric population the data is still scarce, possibly because of the lack of a homogeneous way to assess malnutrition (BMI, serum albumin, serum proteins, arm muscle area, arm fat area…), and considering the challenge of addressing nutritional needs in the different phases of childhood (18). White et al. conducted a single-center retrospective study, dividing patients into three different categories based on pre-transplant weight: underweight, ideal weight and overweight. These categories were determined by identifying the patient’s height percentile and selecting the ideal body weight based on the corresponding weight percentile. A child was defined as underweight if the ratio between his body weight and the ideal body weight was lower than 0.9 and overweight if the ratio was higher than 1.1. Children who were overweight before allo or auto HSCT have a reduced probability of survival compared with ideal-weight children (hazard ratio 1.91; 95% confidence interval, 1.10–3.31). No significant increase in mortality was observed in underweight patients (30). On the other side, in children and young adults diagnosed with acute leukemia who underwent umbilical cord blood transplantation, a BMI less than the fifth percentile at the time of transplantation was associated with higher incidence of acute grade II to IV aGvHD (31, 32). In the auto HSCT setting, a BMI <5th percentile at the time of transplantation was significantly associated with increased incidence of electrolyte disorders, mainly hypokalemia, and severe mucositis (33). Other conflicting data are reported by Aplenc et al. who retrospectively analyzed 3,687 children from the Center for International Blood and Bone Marrow Transplant Research (CIBMTR) database receiving cyclophosphamide-based conditioning regimens for leukemias. They found that BMI pre transplant was not significantly associated with different survival after allo HSCT. Obese children experienced less relapse compared with patients with normal BMI but this benefit was offset by increased transplant related mortality (34). Considering the aforementioned limits of weight and BMI as indicators of nutritional status, other anthropometric measures could be assessed to evaluate the relationship between nutritional status and clinical outcomes. Hoffmeister et al. evaluated mid-upper arm circumference and triceps skin fold thickness pre transplant and observed that arm muscle area <5th percentile was associated with lower event free-survival, higher non-relapse mortality and relapse rate at day 100 and 3 years post allo-HSCT, while BMI 5–24th percentile and arm fat area <25th percentile were associated only with short term outcomes and were not predictor of 3 years outcomes (35). Among several biomarkers, albumin is the only one that has been studied in the pediatric HSCT setting. Children with hypoalbuminemia prior to transplant had increased need for critical care intervention, with higher rates of non-invasive and invasive ventilation and vasoactive therapy. Moreover, these patients had a higher 6-months mortality (36). In another study, serum albumin level ≥3 mg/dL on day 5 after the start of steroid therapy for gut aGvHD and response to steroids were significantly associated with a reduced non-relapse-mortality and an increased overall survival (37). Considering that single nutritional parameters present several limits in predicting outcome, composite nutritional risk scores has been created in order to better unravel the clinical impact of nutritional status. Kerby et al. addressed this issue by composing two variables called NUT25 and NUT5, defined as any of the following: albumin <2.8 g/dl, weight loss ≥10% from baseline, and BMI <25th or <5th percentile, respectively. These markers were assessed pre transplant and every 30 days in the first three months after allo-HSCT. Both low BMI and NUT25 and NUT5 at any time point predicted an increased risk of developing grade III–IV aGvHD in the subsequent 30 days even after adjusting for other risk factors. Moreover, NUT25 at baseline was associated with increased 100-day mortality (27). Additional evaluation of nutritional status can be performed through the analysis of micronutrients, among which the best studied is Vitamin D. Interestingly, hypovitaminosis D pre HSCT was associated with reduced overall survival and increased relapse rate in children with malignancies, and with slower recovery of neutrophil granulocyte counts (38) (Table 1). Nutritional status also has a profound impact on a key predictor of outcomes in the allo-HSCT setting: the gut microbiome (GM) (39–43). Nutritional status and GM have a bidirectional relationship. Disturbances in the microbiome affect the risk for undernutrition and obesity through the alteration of bacterial metabolites production, and malnutrition alters GM function and composition (44–46). To date, no study in the pediatric setting has been completed, but promising data on preclinical and clinical adult models shows a complex interplay between obesity, sarcopenia, the GM and its metabolome, that could have key repercussions on clinical endpoints (29, 47).

**TABLE 1:** Main studies on the relationship between nutritional status and clinical outcomes in children.

References	Clinical setting	Nutritional status measurement	Clinical finding
White et al. (30)	Auto- and Allo-HSCT	Pre-transplant weight	Overweight have reduced probability of survival compared with ideal-weight. Underweight no increase in mortality.
Aplenc et al. (34)	Allo-HSCT with cyclophosphamide-based conditioning for acute leukemia	BMI pre-transplant	Not significantly associated with different survival after allo-HSCT. Obese less relapse but increased transplant related mortality
Paviglianiti et al. (31)	Umbilical Cord Blood HSCT in Children and Young Adults with Acute Leukemia	BMI at the time of transplantation	BMI <5th percentile associated with higher incidence of severe aGvHD
Kranjčec et al. (33)	Auto-HSCT	BMI at the time of transplantation	BMI <5th percentile associated with increased incidence of electrolyte disorders, and severe mucositis
Hoffmeister et al. (35)	Allo-HSCT for hematologic malignancies	BMI, arm muscle area, and arm fat area pre-transplant	Arm muscle area <5th percentile associated with lower event free-survival, higher non-relapse mortality and relapse rate at day 100 and 3 years BMI 5–24th percentile and arm fat area <25th percentile associated only with short term outcomes and were not predictor of 3 years outcomes
Teagarden et al. (36)	Allo-HSCT	Albumin levels pre-transplant	Hypoalbuminemia associated with higher 6-months mortality, increased need for critical care intervention, non-invasive and invasive ventilation and vasoactive therapy
Goussetis et al. (37)	Gut aGvHD	Albumin levels on day 5 after the start of steroid therapy	Serum albumin level ≥3 mg/dL significantly associated with reduced non-relapse mortality and increased overall survival
Kerby et al. (27)	Allo-HSCT	NUT25 and NUT5: albumin <2.8 g/dl, weight loss ≥10% from baseline, and BMI <25th or <5th percentile, respectively. Pre-transplant and every 30 days in the first three months	Low BMI and NUT25 and NUT5 at any time point predicted an increased risk of developing grade III–IV aGVHD in the subsequent 30 days. NUT25 at baseline associated with increased 100-day mortality
Hansson et al. (38)	Allo-HSCT	Vitamin D levels pre-transplant	Hypovitaminosis D associated with reduced overall survival and increased relapse rate in children with malignancies, and with slower recovery of neutrophil counts

## Enteral and parenteral nutrition

As previously mentioned, the severe impairment of oral intake in the early post-transplantation period due to the conditioning regimens, intestinal aGvHD, infections and other medications, such as opioids, affect dramatically the nutritional status. Thus, nutritional support is essential in these patients. As previously mentioned, pediatric HSCT recipients should be screened and assessed for impending or overt malnutrition at admission and after that frequently monitored with a comprehensive nutritional assessment. If deficits are observed, nutritional support, including nutritional counseling, oral nutritional supplements, parenteral (PN) and enteral nutrition (EN) should be initiated early to avoid or minimize further weight and muscle mass loss (9, 16, 48). The first form of nutritional support should be nutrition counseling to help manage symptoms and encourage the oral intake of protein- and energy-rich foods and fluids that are well tolerated. The additional use of oral nutritional supplements is advised when diet is not effective in reaching nutritional goals (9, 16). If oral nutrition is not tolerated, as in most pediatric patients in the neutropenic phase, artificial nutrition is usually necessary and is generally indicated when oral caloric intake is below 60–70% of requirements for 3 days. PN and EN are the two main strategies adopted in the transplantation setting in order to provide nutrition when oral nutrition is insufficient (11, 49). PN has been historically considered the method of choice and is still frequently adopted in transplantation centers as the first choice for nutritional support in the early post-transplant period (50). However, PN is associated with several complications, particularly infective and metabolic, with increased direct and indirect costs (51–53). EN is currently recommended as first-line nutritional support in transplant recipients when oral intake is not possible, as highlighted in recent international guidelines (11, 49, 54). PN could still be preferred only in case of intractable vomiting, ileus, severe malabsorption or symptomatic gut GvHD (16). A report from pediatric disease working party of EBMT also specifically recommends early enteral feeding as the first option in children undergoing HSCT (55). The benefits of EN include the maintenance of mucosal gut integrity and barrier, the stimulus to mucosal repair with decreased risk of infections and hyperglycemia and lower costs compared to PN (56, 57). Even though, these recommendations are based on weak evidence with a lack of randomized clinical trials (14). This is one of the reasons for the variability in nutritional approaches among transplant centers (50, 58). Other barriers to EN implementation include gastrointestinal and oral distress, possible hematological and electrolytic complications and institutional practices (56). Furthermore, specific considerations in the pediatric population include the importance of growth and development with more pronounced consequences of inadequate nutrition and the lack of patient autonomy (59–61). A systematic review published in 2019 compared EN vs. PN in pediatric HSCT reporting conflicting results about nutritional and clinical outcomes; a favorable effect was provided by EN over PN regarding aGvHD, with a lower incidence of grade III-IV aGvHD in EN groups in two studies (62, 63). Meta-analysis was not performed due to the heterogeneity of the four included studies regarding populations, interventions and outcomes analyzed (64). Faster platelet engraftment was found in the EN group in two studies, possibly secondary to the lower incidence of aGvHD-related thrombocytopenia (62, 63). Interestingly, one study found better early outcome in the EN group compared to the PN group, with lower mortality rate and non-relapse mortality rate (62). This finding was consistent with other reports in adult HSCT, but further studies are surely necessary in the pediatric population (62). It is important to underline that EN was generally well tolerated, confirming the feasibility of this approach in the pediatric population (51, 62, 63, 65, 66). A recent meta-analysis analyzed both pediatric and adult HSCT recipients comparing EN to PN and confirmed lower incidence rates of aGvHD, grade III-IV aGvHD and intestinal aGVHD in EN groups, including patients who received EN as primary nutritional support with or without the addition of PN; no differences were demonstrated regarding the incidence of oral mucositis and overall survival. Data regarding infectious complications and hematological recovery were inconclusive (67). Therefore, the relationship between nutritional support and aGvHD has largely been demonstrated in several reports (62, 63). Increased mucosal atrophy due to conditioning regimen toxicity together with the complete resting of the gut due to PN affects GM homeostasis (68). On the other hand, EN acts with a trophic effect on the mucosa, ensuring gut barrier function and reducing bacterial translocation with a beneficial effect on gut bacterial composition (57, 69, 70). Two studies specifically investigate the effect of EN and PN on microbiota, suggesting a possible modulation of gut bacterial populations by nutritional interventions (58, 68). A recent report found interesting results comparing EN to total PN confirming the protective effect of EN on aGvHD risk and also reporting a significant lower rate of sinusoidal obstruction syndrome in the EN group, possibly due to the impairment of liver and biliary function from PN (71). The possible are use of gastrostomy to give EN in children undergoing HSCT was also assessed, founding this approach feasible with lower rate of PN requirement (72). In a recent survey, authors highlighted the importance to weigh potential benefits against risks of gastrostomy placement in these high-risk population and future studies about safety and long-term outcomes are certainly needed in order to make final recommendations, also considering family preferences and perceptions (73).

## Diet post-transplant

The type of diet to administer after HSCT, both during the hospitalization and after discharge, represents a long-lasting debate among pediatric oncologists. Starting from the early 1960s, patients were used to receive autoclaved sterile food to keep them in a bacterial-free environment. Moreover, patients also received gut decontamination with neomycin, polymyxin B, cephaloridin and amphotericin B to completely sterilize the intestine (74, 75). This was ideated following the concept that aliments contain bacteria and thus represent a risk for food-borne infections in immunocompromised patients. In addition, gut decontamination was historically correlated, in preclinical model to a reduced risk of developing GvHD. However, the very low palatability of the food advocated for a change, thus the so-called neutropenic diet (ND) was developed. It consists of a particular diet in which all the contaminated foods were excluded (76). In particular, fresh fruits and vegetables were excluded and dairy and meat products were strongly limited (77). Notably, no randomized trials ever demonstrated a proven reduction in the risk of infectious complications for ND. Moreover, often these diets are difficult to follow, not homogenously performed and carry the risk of nutritional deficiencies and inadequate food intake, especially in pediatric patients with selective eating. Despite these difficulties, ND has been largely administrated to pediatric and adult patients on a precautional basis, as demonstrated by several surveys (50, 78, 79). Only recently, some evidence has questioned the usefulness of this kind of diet also pointing to a detrimental effect on the GM (80). As a matter of fact, diet is a strong modulator of the GM and a rich and diverse GM has been associated with better clinical outcomes in both pediatric and adult patients (39–41). A recent meta-analysis on adult and pediatric non-HSCT cancer patients showed no differences in terms of infection and mortality rate between ND and a control diet (80). In the HSCT setting, observational studies, at first, and then randomized controlled trial in adult patients confirmed that the ND does not confer a clinical benefit (81). In 2009 the CIBMTR, on the basis of this evidence, reduced the list of food to be avoided, also introducing the possibility to eat fast food (82). Food deemed difficult to clean, such as some kind of fruits and vegetables, was still recommended to be avoided (82). Studies directly addressing the role of ND in pediatric patients receiving HSCT are only a few. Taggart et al. reported a controlled trial on pediatric patients receiving HSCT comparing clinical outcomes before and after the shift from ND to a food safety-based diet. The latter policy allowed the patients to assume all fresh raw fruits and vegetables, previously washed under running water and free from visible damage. Authors showed no differences in the two groups in terms of systemic infections, GvHD, and death in the first 100 days (83). In view of the abovementioned evidence, the Pediatric Diseases Working Party of the European Society for Blood and Marrow Transplantation (EBMT) suggests replacing the ND with safe food handling guidelines (84). These recommendations are based on four mainstays: Clean, separate, cook, and chill and consist of several precautions in food handling to reduce pathogens overgrowth in the food. Even if recommendations are growing, no clear evidence is available and is thus highly awaited. One reason that might explain the lack of benefit for ND is that bacteria found on fresh fruits are part of the normal GI flora and do not carry pathogenic potential (77, 85). Probably, more in-depth studies will help to uncover the role of food bacteria in the context of GM homeostasis and to define the proper food choice and handling. Diet post-transplant should also be studied considering the changes of body composition that usually occurs. In details, several evidence has shown that pediatric and young adults receiving HSCT experience a remodeling of adipose tissue earlier than peers toward lipodystrophy and a reduction in muscle mass (86). Evidence of the effect of specific diets on these alterations are missing and should be investigated in the future.

Interestingly, several cases of newly onset food allergy after HSCT, particularly cord blood transplantation, have been reported (87, 88), with symptoms ranging from urticaria, angioedema, diarrhea and vomiting to eosinophilic gastrointestinal disorders or even anaphylaxis (87, 89, 90). This may be partially explained by immune reconstitution toward a Th2 response in this setting, probably favored by calcineurin inhibitors (87). Symptoms compatible with food allergies should therefore be taken into account in dietary management after HSCT.

## Dietary compounds

The importance of EN in the setting of pediatric allo-HSCT has been increasingly recognized in order to preserve a condition of GM eubiosis, thus reducing the risk of GVHD and infections (70, 91). Traditionally, moreover, traditionally certain dietary compounds have been widely restricted to reduce the risk of food contamination for the HSCT recipient following ND, which is increasingly questioned (84) as explained above. However, less is known about the impact of specific dietary compounds on HSCT outcomes. Emerging evidence about the complex interaction between dietary compounds and GM may guide the choice of the optimal enteral supplementation strategy. In adults, strong evidence of the negative impact of lactose during allo-HSCT has been reported by Stein-Thoeringer et al. (91). Lactose in the gut lumen drives fecal *Enterococcus* spp. domination, which was associated with a significant reduction in overall survival and an increased risk of moderate-to-severe aGvHD. This finding assumes a great relevance because epithelial damage of the gut mucosa induced by chemotherapy or intestinal GvHD are common findings during allo-HSCT. This leads to a secondary lactase deficiency and ultimately to increased levels of lactase in the gut lumen. Since this data are gathered from a cohort of adult patients from adults, we have to consider the difference in lactase activity in children before translating them in the pediatric setting. In fact, lactase activity is high in all fully mature human babies, then the persistence of lactase activity is determined by a genetic polymorphism of the gene encoding for the lactase enzyme located on chromosome 2 (MCM6) (92, 93). Three main genotypes are recognized: lactase persistent (LP, allelic variant T/T 13910), lactase non-persistent (LNP, allelic variant C/C 13910) and heterozygotes (allelic variant C/T 13910). Being LNP is the most widespread allelic variant (65–70% of the population), lactase activity is assumed to be lower in adults (92).

Glutamine is an essential amino acid that is an essential nutrient for some cells, such as enterocytes and lymphocytes, and might minimize the intestinal damage associated with conditioning regimens of allo-HSCT. Several studies evaluated the impact of glutamine on allogeneic HSCT outcome (94–96), and glutamine was also associated with beneficial effects in meta-analysis (97). Oral glutamine might reduce mucositis and GvHD, whereas intravenous glutamine might reduce the risk of infections. Recent large randomized controlled trials suggested a detrimental effect of glutamine administration in critically ill patients (98, 99). Although the precise mechanism of action remains unclear, glutamine may lead to amino acid overload in patients with renal impairment (98). Gjaerde et al. reported that high vitamin E levels prior to transplantation were associated with less grade II–IV acute GvHD after myeloablative allogeneic HSCT (100). This may be due to the immunomodulatory properties of the vitamin E which might inhibit the release of reactive oxygen species and pro-inflammatory cytokines by innate immune cells (101) which drives the early phase of acute GvHD in the gut (102). Vitamin E also prevents the adhesion of immune cells to the endothelium (103). Lactoferrin has been recently tested in pediatric patients receiving induction chemotherapy with a positive effect of the GM and future studies are warranted to confirm its role also in the HSCT context (104). An interesting case was reported of the successful treatment with oral lactoferrin of gut aGvHD refractory to conventional immunosuppressive therapy, highlighting its potential in this setting (105). The immunomodulatory activity of Omega-3 and other PUFA (Poly Unsaturated Fatty Acids) has been increasingly recognized in children in various settings such as autoimmune diseases, inflammation and food allergies (106–108). PUFA have the potential to modulate both innate and adaptive immunity (109, 110), with specific mechanisms of action. Eicosapentaenoic acid (EPA) and Docosahexaenoic acid (DHA) derived mediators, namely resolvins, protectins, and maresins, appear involved in inflammation resolution (111, 112), but data in children remains scarce. Furthermore, EPA and DHA seem to possess a more powerful immunomodulatory activity than linolenic acid (ALA) (106). In the setting of allogeneic HSCT, Omega-3 might mitigate the cytokine storm, possible contributing to reducing complications. Takatsuka et al. reported that oral Omega-3 administration could help mitigate GvHD severity in a small cohort of young adults (113).

The complex interplay between the GM and the implementation of prebiotics (114, 115) and probiotic (116, 117), compounds in the allogeneic HSCT recipients diet showed some potential benefit on various outcomes in children and adult patients (Table 2). Furthermore, preclinical evidence of the potential role of Synbiotic (118) and postbiotic (119, 120) compounds are growing, but more interventional clinical trials are needed to confirm this data. Despite the evidence on specific dietary compounds in the pediatric population remains scarce, the literature on adults indicates that nutritional modulation of the GM is an expanding field of research to improve outcomes for children undergoing allo-HSCT.

**TABLE 2:** Main studies on gut microbiota modulation in allo-HSCT.

References	Dietary compound	Setting	Effect on GM	Effect on outcomes
Stein-Thoeringer et al. (91)	Lactose	Adults, allo-HSCT	*Enterococcus* spp. domination	Reduction of overall survival and increase of aGvHD
Iyama et al. (114)	Glutamine, fiber and oligosaccharides	Adults, allo-HSCT	*Enterococcus* species translocation reduction	Increased overall survival and decreased severity of intestinal mucositis
Yoshifuji et al. (115)	Resistant starch and prebiotics containing glutamine, polydextrose, and lactosucrose	Adults, allo-HSCT	No difference in GM composition and diversity.	Shortened duration of oral mucositis and diarrhea. Reduction of cumulative incidence of grade II-IV aGvHD
Ladas et al. (116)	*Lactobacillus plantarum*	Children and adolescent, allo-HSCT	GM colonization with administration of *Lactobacillus plantarum*	No cases of *Lactobacillus plantarum* bacteremia.
Gorshein et al. (117)	*L. rhamnosus GG*	Adults, allo-HSCT	GM composition wasn’t affected by supplementation.	No difference in the incidence of GvHD.

## Conclusion

Malnutrition is a common feature of pediatric patients undergoing HSCT with the detrimental effect of clinical outcomes and GM. Nutritional support could be considered a risk- and cost- effective way to improve allo-HSCT outcomes in children. However, many questions remain to be answered and this is currently limiting the diffusion of specific programs for nutritional support. First, while evidence on adult patients is consistent, data on pediatric patients are only few and more specific studies are warranted. Moreover, an in-depth analysis of how nutritional assessment should be performed could improve clinical evaluation and provide stronger data on the correlation with clinical outcomes. Safety concerns are also limiting the experimentation on new dietary compound representing an important point of focus. We believe that a multidisciplinary team composed of pediatric hematologists, gastroenterologists, nurses, physiotherapists and dieticians should carry on a structured nutritional evaluation during HSCT and should design the best nutritional support for each patient. A radical change has been seen in this regard in recent years, with the reduction of ND, the rise of EN and the administration of specific molecules potentially modulating GM. This novel nutritional platform opens up great opportunities to improve nutritional support, and consequently clinical outcomes.

## Author contributions

EM, DL, FB, FV, SC, and AZ wrote the manuscript. DL and SC designed Figure 1. ML, AP, RM, and DZ critically reviewed the manuscript. All authors contributed to the article and approved the submitted version.
